# Acceptability of breast cancer risk assessment amongst general population women aged 30–39 years: A qualitative study

**DOI:** 10.1177/17455057261435107

**Published:** 2026-04-24

**Authors:** Rosie Horton, Sarah Hindmarch, Sacha J. Howell, Juliet A. Usher-Smith, Louise Gorman, D. Gareth Evans, David P. French

**Affiliations:** 1 Manchester Centre for Health Psychology, Division of Psychology and Mental Health, School of Health Sciences, Faculty of Biology, Medicine and Health, University of Manchester, UK; 2 Manchester Academic Health Science Centre, Division of Cancer Sciences, Faculty of Biology, Medicine and Health, University of Manchester, UK; 3 Primary Care Unit, Department of Public Health and Primary Care, University of Cambridge, UK; 4 NIHR Greater Manchester Patient Safety Research Collaboration, Division of Population Health, Health Services Research & Primary Care, Faculty of Biology, Medicine and Health, University of Manchester, UK; 5 Manchester Academic Health Science Centre, Division of Evolution, Infection and Genomic Sciences, School of Biological Sciences, Faculty of Biology, Medicine and Health, University of Manchester, UK; 6 St Mary’s Hospital, Manchester University NHS Foundation Trust, Manchester Academic Health Science Centre, North West Genomics Laboratory Hub, Manchester Centre for Genomic Medicine, UK

**Keywords:** breast cancer, risk assessment, acceptability, risk communication, qualitative research

## Abstract

**Background:**

Breast cancer rates are increasing globally amongst premenopausal women, who often face aggressive subtypes with poorer outcomes than postmenopausal women. In the United Kingdom, current guidelines for risk assessment amongst women under age 50 years focus on family history or genetic predisposition, a strategy that fails to identify most women that go on to develop breast cancer. The Breast CANcer Risk Assessment in Younger women (BCAN-RAY) study is evaluating the feasibility and acceptability of a novel risk assessment strategy for women aged 30–39 years, without a strong family history, incorporating a risk factor questionnaire, polygenic risk score and breast density assessment to identify those at increased risk and support early screening and preventive health measures.

**Objectives:**

This study aimed to investigate the acceptability of the BCAN-RAY approach to assessing risk by exploring women’s views of the invitation, risk assessment and feedback processes used, to identify any changes required.

**Design:**

A cross-sectional qualitative design was used.

**Methods:**

Twenty-two women from the BCAN-RAY study identified as being either at population average (*n* = 11) or at increased risk (*n* = 11) of developing breast cancer completed semi-structured interviews shortly after receiving risk feedback. Data were analysed thematically.

**Results:**

Participants generally found the BCAN-RAY approach acceptable, with no evidence of significant anxiety during or after participation. Positive experiences were largely attributed to personalised staff interactions and the availability of tailored opportunities to manage risk. However, some participants reported confusion regarding risk information provided, for example, struggling with technical language. Practical barriers, including logistical challenges and limitations of existing referral pathways, were also identified.

**Conclusion:**

Breast cancer risk assessment amongst younger women was generally acceptable with results allowing participants to be proactive about their health. Issues related to risk communication, training of staff who will conduct risk assessment and streamlining the delivery of risk assessment need to be considered for routine implementation.

## Introduction

Over the past decade, breast cancer incidence has been increasing in premenopausal women worldwide.^1–4^ This trend is concerning; as compared to postmenopausal women, younger women are more likely to develop aggressive breast cancer subtypes, which are associated with higher recurrence and poorer survival despite intensive treatment strategies.^5,6^ In the United Kingdom, breast cancer is the most common cause of death in women aged 35–49 years.^7^ These women may have decades of quality life years to lose and very often have young families. There is, therefore, a growing urgency to implement initiatives to identify younger women at increased risk such that early screening and National Institute for Health and Care Excellence (NICE) approved preventive strategies including risk-reducing medication can be offered.^8^

The development of breast cancer risk prediction models has led to improvements in the estimation of a woman’s risk of developing breast cancer with sufficient levels of accuracy.^9–11^ In the United Kingdom, a family history of breast cancer or known high-risk germline mutation in a close relative is the only criterion by which women aged under 50 years can access screening and preventive strategies through referral to family history clinics.^12^ However, at least 65% of women who develop breast cancer before the age of 50 years do not have such a family history known and are not prospectively identified as being at increased risk.^13,14^

In recent years, additional breast cancer risk factors have been identified including reproductive (e.g. age of first full-term pregnancy) and hormonal history (e.g. age of menarche), polygenic risk scores and mammographic density. Incorporating these additional risk factors into risk prediction models has been found to result in improved discriminatory accuracy across all age groups including women aged under 50 years.^15–18^ The provision of personalised breast cancer risk estimates has predominantly been investigated within the context of implementing a risk-based breast cancer screening programme.^19^ Recruitment is ongoing for international trials to determine the effectiveness of a risk-based approach at preventing development of later stage breast cancers via uptake of more frequent screening and preventive strategies in women identified as being at increased risk.^20,21^ However, inclusion of breast cancer risk assessment at the time of national mammographic screening programmes will miss younger women eligible for screening and preventive strategies. Consequently, there is a growing interest in offering comprehensive breast cancer risk assessment from an earlier age.

The Breast CANcer Risk Assessment in Younger women (BCAN-RAY) study (NCT05305963) in Greater Manchester aimed to evaluate a comprehensive breast cancer risk assessment strategy amongst a population of women aged 30–39 years, from diverse ethnic and socio-economic backgrounds, and without a strong family history of breast cancer.^22^ The primary objective of the BCAN-RAY study was to quantify the risk associated with breast density in this age group. To address this, we have developed a low-dose mammogram technique, which uses one tenth or less of the radiation dose of a full-dose screening mammogram making it safer, which has been shown to be accurate in younger women.^23^ The risk assessment strategy thereby consists of a questionnaire of breast cancer risk factors, a saliva sample to assess polygenic risk and the presence of pathogenic variants in high- and moderate-risk genes, and low-dose mammography to measure breast density. The breast cancer risk assessment strategy adopted in the BCAN-RAY study is herein referred to as the BCAN-RAY approach.

In line with the Medical Research Council Framework for Developing and Evaluating Complex Interventions,^23^ one of the objectives of BCAN-RAY is to assess the feasibility and acceptability of the BCAN-RAY approach. Detailed information about the planned methodology and analyses can be found in the feasibility study protocol.^24^ It is important to consider acceptability to optimise the likelihood of future implementation at a population level being successful. If the processes of invitation, risk assessment and feedback are unacceptable, then the uptake of breast cancer risk assessment will be negatively impacted.

Previous research has explored the acceptability of women having their risk assessed as part of routine breast screening and receiving breast cancer risk estimates.^25,26^ Overall, women found it acceptable and valuable to be offered breast cancer risk assessment as part of screening. However, there is a need to establish acceptability amongst younger women for several reasons. First, one might expect different levels of distress amongst younger women at increased risk as the result may be more unexpected because of a lack of family history of the disease. Second, due to the potential implications of being identified as at increased risk of younger women in terms of reproductive decision-making, younger women may have different reactions to taking part in breast cancer risk assessment than older women. We have previously conducted a qualitative study with women aged 30–39 years, which suggested that undergoing breast cancer risk assessment was acceptable in principle.^27^ However, risk assessment was presented as a hypothetical prospect in that study, so how women may respond once they have experienced it remains unknown.

The present study aimed to investigate the acceptability of the BCAN-RAY approach by exploring women’s views and experiences of undergoing breast cancer risk assessment and receiving a breast cancer risk estimate, shortly after participation. Specifically, we wanted to explore women’s views on the invitation, risk assessment and feedback processes including provision and receipt of information and support, and suggestions for any changes to the offer of breast cancer risk assessment to increase engagement and uptake in future.

## Methods

### Design and participants

Ethics approval was granted by North West – Greater Manchester West Research Ethics Committee (reference: 22/NW/0268), and the study was performed in accordance with the Declaration of Helsinki, Good Clinical Practice principles and relevant regulations. The reporting of this study conforms to the COREQ statement.^28^

This qualitative study was nested within the wider BCAN-RAY case-control study. Approximately 1000 women were recruited to BCAN-RAY between May 2023 and May 2025: 250 women diagnosed with breast cancer when they were aged 30–39 years (cases) and 750 women currently aged 30–39 years without a strong family history of breast cancer (controls). In this study, strong family history was defined as a first-degree relative diagnosed with breast cancer under the age of 50 or two or more second-degree relatives diagnosed with breast cancer at any age.^24^ Women with germline mutations were ineligible to participate because they can access screening and preventive strategies through referral to family history clinics. Detailed BCAN-RAY inclusion and exclusion criteria are provided in the feasibility study protocol.^24^

In the present study, women were eligible if they had participated in the control arm of BCAN-RAY, received their 10-year risk of breast cancer feedback, and could communicate in English. Participants were excluded if they were unable to conduct the interview in English or did not consent to the interview being audio-recorded. A cross-sectional design was adopted employing one-to-one semi-structured interviews. This method enables the participant to be open with their responses and allows the researcher to capture in-depth accounts of the experience under study.

### BCAN-RAY study procedure

Women were invited to take part in the BCAN-RAY study via letter from participating general practices. The risk assessment process involved completing a self-report risk factor questionnaire on a bespoke web-based application, providing a saliva sample to assess polygenic risk and the presence of pathogenic variants in high and moderate-risk genes, and undergoing low-dose mammography to measure breast density.^29,30^ Results from all risk components are uploaded into the CANRISK programme, producing a 10-year breast cancer risk estimate using the BOADICEA algorithm.^31^ Women were given one of two risk category estimates based on their predicted 10-year risk of breast cancer at age 40 (population average (<3%) or increased (⩾3%) in line with NICE moderate-risk threshold for early intervention guidelines facilitating access to screening and preventive strategies in the latter group).^12^ The NICE risk categories are near population, moderate and high. The decision to adopt average and increased only was informed by findings of a qualitative study we conducted with women who matched the intended recipients of the BCAN-RAY study.^27^ Women suggested the use of high would be too alarming for the initial notification of risk result as at this stage participants would be able to access support immediately. Instead, we adopted ‘average’, which was equivalent to near population and ‘increased’ to capture both moderate and high groups. No margins of error or levels of accuracy were provided to participants at average risk and white British women at increased risk. During the risk review appointment, non-white British women were informed of the relative inaccuracy of the risk prediction based on what is known about differential performance of polygenic risk scores amongst ethnic minority groups and told they would receive updated risk feedback next year once an adjustment for ancestry has been applied. Women received a risk feedback letter within 16 weeks of completing the risk assessment informing them of their risk category (see Supplemental Material 1), and the full CANRISK report providing more detailed information including lifetime risk and comparison with population risk statistics.^32^ The CANRISK report was co-designed with patients, the public and healthcare professionals, and this process included an assessment of its comprehensibility.^32^ Leaflets detailing ways of reducing breast cancer risk, signs and symptoms of breast cancer and breast awareness were also provided. Those identified at increased risk were offered a telephone appointment with an experienced breast oncologist (SJH) with expertise in risk assessment, screening and prevention, working in a family history clinic. At this appointment, the option to initiate annual breast screening, when the 10-year risk met the 3% threshold, and preventive measures including risk-reducing medication were discussed. Upon completion of recruitment and analysis, breast density will be incorporated into individual risk assessments and ancestry adjustments applied for non-white British women. This updated feedback will be provided to participants, and this may result in some women switching risk categories.

#### Qualitative study procedure

As part of the BCAN-RAY study, participants were also asked to complete three questionnaires, with the first questionnaire completed following the submission of the risk factors questionnaire online but before the saliva sample was collected and the low-dose mammogram performed.^24^ At the end of this questionnaire, participants indicated whether they were willing to be contacted about participating in an interview regarding their experience of breast cancer risk assessment following the receipt of their risk feedback letter.

A sample of average and increased risk women who agreed to be contacted were sent an invitation to take part in an interview, and a participant information sheet, via e-mail or letter, in line with their recorded contact preference. A purposive sampling approach was adopted whereby self-reported demographic characteristics and responses to the baseline psychological impact questionnaire guided sampling to maximise diversity of the sample in terms of ethnicity, socio-economic status (using residential postcode as a proxy measure), and anxiety levels of participants. Demographic information was obtained from the BCAN-RAY main study database. Women who received an average risk estimate were invited approximately 1 month after they received their risk feedback from the BCAN-RAY study team. Women who received an increased risk estimate were invited approximately 3 months post-risk feedback once they had received the risk review telephone appointment. This allowed women to explore additional screening and risk-reducing strategies prior to the interview, minimising any influence participating in the interview may have on decision-making. Those interested in taking part were asked to contact the research team by e-mail or telephone. If no response was received following the initial invitation, a second invitation was sent approximately 3–4 weeks later.

Interviews took place between January 2024 and January 2025, either face-to-face at a university building, in participant’s homes or via telephone according to participants’ preferences. Consent was obtained prior to any study procedures. Written consent was obtained for face-to-face interviews. Audio-recorded verbal consent was obtained for telephone interviews using a consent script. Interviews were audio-recorded and transcribed verbatim by an external transcription company. Personally identifiable information was anonymised, and participants were assigned pseudonyms. The decision to cease recruitment was guided by the concept of ‘information power’. The research team reflected on the information richness of their dataset throughout data collection to determine when sufficient data had been collected to answer the research question.^33^ A £20 shopping voucher was offered to participants in recognition of their time given to the study.

### Topic guide

Topic guide development was informed by the aims of the study and an evidence-based framework of acceptability.^34^ In line with this framework, we defined acceptability as the extent to which women considered the BCAN-RAY approach to be appropriate, based on experienced cognitive and emotional responses to participating in risk assessment and receiving risk feedback. An initial draft was developed by SH, a health psychology researcher with qualitative health services research experience. Feedback on this draft was obtained from public contributors and members of the research team (DPF and JAU-S) who have research expertise in breast cancer and screening services, primary care and health services research, health psychology and qualitative methods. The content and structure of the topic guide were revised in line with the feedback received. Questions explored women’s experience of participating in breast cancer risk assessment and receiving a breast cancer risk estimate, views on the invitation and risk feedback materials and how the risk assessment process could be improved in terms of delivery/access and provision of information and support (see Supplemental Material 2). Furthermore, women were asked to discuss any actions they had considered and/or made as a result of participating in breast cancer risk assessment (e.g. health-related behaviour change, additional screening and risk-reducing medication). The topic guide was followed flexibly, allowing participants to raise and discuss issues important to their own experiences of receiving breast cancer risk assessment and risk feedback.

### Patient and public involvement

A public involvement group of 11 women aged 30–39 years was established in September 2021 to inform the development of research, including the BCAN-RAY study, aimed at identifying young women at increased risk of breast cancer. Different women from this group completed different tasks for the study. Two women reviewed the study documentation (participant information sheet, invitation letter and interview topic guide). For all documents, they provided feedback on the clarity of information, which included suggestions for changes to wording and structure. For the interview topic guide, they commented on the relevance and scope of questions including suggestions for additional questions and prompts, and changes to the order of questions. Study documentation was revised in line with the feedback. The study findings were presented to two members of the public involvement group. They were asked to consider the implications of said findings for improving the provision of breast cancer risk assessment in the future (see Supplemental Material 3 for further detail).

### Data analysis

Data were coded within the NVivo14 software (*Lumivero*) using a manifest level approach to reflexive thematic analysis.^35,36^ Reflexive thematic analysis was chosen because this method is particularly suitable when the aim is to explore participants’ experiences and sense-making,^37,38^ and this aligned well with the aim of the present study to explore women’s views and experiences of undergoing breast cancer risk assessment and receiving a breast cancer risk estimate. A critical realist approach was taken, meaning the researchers accepted that participants’ accounts represent their perception of their reality, which is shaped by and embedded within their cultural context and language.^39^ An experiential orientation to data interpretation was adopted that aimed to stay close to participants’ meanings and capture these in ways that might be recognisable to them.

Throughout the analysis, reflexivity was maintained by the research team to ensure that their own perspectives and biases were acknowledged and minimised. Interviews were conducted by RH and SH. Both interviewers are female researchers with backgrounds in psychology and experience in qualitative research. RH has had no previous experience in cancer prevention and early detection. In contrast, SH has worked in cancer prevention and early detection research for 9 years, both reflecting and shaping her favourable opinions of early cancer detection initiatives. Regular team meetings were held to discuss and validate the themes, in order to increase the credibility and trustworthiness of the findings.

## Results

A total of 101 women were invited to take part in the study. Twenty-five women contacted the research team expressing an interest in the study. Two women did not respond to the research team following two contact attempts, and one woman declined participation stating she no longer had the time to take part. Therefore, 22 women were interviewed, 11 at increased and 11 at average risk (see Table 1). Interviews ranged from 20 to 79 min (median = 36 min). Sixteen of the 22 women identified as white British. Five women interviewed were from more deprived locations (deprivation decile ⩽3). For increased risk women interviewed, risk scores ranged from 3% to 6.3% (mean = 4.1%).

**Table 1.** Demographic characteristics of sample interviewed (*n* = 22).

Characteristic	*N* (%)
Risk result	
Average	11 (50)
Increased	11 (50)
Age range (years)^a^	
30–33	6 (27)
34–36	4 (18)
37–39	12 (55)
Ethnicity	
White British	16 (73)
Chinese	1 (4.5)
Indian	1 (4.5)
White (other)	1 (4.5)
Mixed (white/black Caribbean)	1 (4.5)
Mixed (other)	1 (4.5)
Other (Arab)	1 (4.5)
Deprivation decile^b^	
High (1–3)	5 (23)
Medium (4–7)	2 (9)
Low (8–10)	15 (68)

a Age at the time of participation in breast cancer risk assessment.

b Assessed by participant residential postcode using the Index of Multiple Deprivation 2019, a measure of relative deprivation for small areas in England.^40^

Data are presented as two overarching themes with four subthemes (Table 2). Quotes are presented with pseudonyms for participants and their risk category.

**Table 2.** Overview of final themes developed.

1. Acceptability of the risk assessment process	1.1. The role of staff support – the personal touch 1.2. Risk assessment enabling a proactive approach towards risk management
2. Improving the offer of breast cancer risk assessment	2.1. Streamlining delivery of breast cancer risk assessment 2.2. Addressing knowledge deficits

### Acceptability of the risk assessment process

Women found the risk assessment process to be acceptable, reporting no concerns with its components and considering their participation worthwhile. Positive experiences were largely attributed to personalised staff interactions, where individuals felt recognised beyond mere numbers, and to the availability of tailored opportunities to manage risk. During interviews, women were asked whether they experienced any anxiety during or after participation in risk assessment. Overall, reports of anxiety were very few, with any anxiety being transient in nature. Women at average risk expressed relief and reassurance, while those identified as increased risk initially experienced mild surprise and concern, which was quickly mitigated through the risk review appointment.

#### The role of staff support: the personal touch

Participants overwhelmingly reported positive experiences with staff members (research practitioners and the research administrator) delivering risk assessment procedures, who were described as professional, competent, and empathetic. These interactions helped reduce anxiety about the risk assessment process, especially the mammogram which none of the women had experienced previously. One participant highlighted, ‘I feel like a personal service, I didn’t feel like I’m a number, you know what I mean, or a city, it wasn’t like that, it was personal touch and was, yeah, it was very good experience within very strange environment’ – Megan, Average Risk.

The role of the research practitioners in supporting women’s understanding of risk assessment was central in helping participants feel comfortable and informed during the risk assessment process, while also providing some clarification on the process itself and next steps, which some women had missed in their written correspondence.

I think the nurse made it more clear what they are going to do, but kind of like I mean initial I knew, it was something related to breast cancer, they are going to do some sort of testing for me. – Sana, Increased Risk

Participants appreciated the research practitioners’ efforts in explaining procedures and addressing concerns, which contributed to their overall positive experience. As one participant noted, ‘that first meeting with the lady in the hospital was just great [. . .] I think it was really well explained, going into that detail was really helpful. Not that I wasn’t clear about it beforehand, but just having it explained again, just to be completely transparent, I thought was really good’ – Vicky, Average Risk.

Similarly, for increased risk women, the risk review appointment was a very positive experience. Many participants expressed feeling more confident in their understanding of their risk and felt that the breast oncologist (SJH) provided clear guidance on how to manage risk and answered all questions kindly and compassionately.

It was explained really clearly and he [breast oncologist] was really just sort of like reassuring and practical and just gave me the information and just kind of the level that was helpful for me, I think. – Jade, Increased Risk

#### Risk assessment enabling a proactive approach towards risk management

All participants valued the opportunity to find out their risk. This information was perceived as beneficial for all irrespective of risk result but particularly so for increased risk women who could access additional healthcare services such as early screening as a result.

I’m really pleased to have received the information and be better informed about my own personal risk [. . .] now I’m eligible for mammograms at forty and that just all feels really positive that there’s sort of like – there’s extra screening in place for me now that I wouldn’t have had access to previously, I think that’s why it feels like it was really worthwhile having gone through that. – Leah, Increased Risk

Many women regardless of risk status had considered adopting healthier behaviours such as altering their diet or increasing their physical activity upon receiving their risk assessment results. Women in both risk categories also expressed heightened breast awareness following participation in BCAN-RAY.

I would definitely say I’m definitely more breast aware now, [. . .] I would definitely say that I do the checks more regularly, not just when I think there’s, you know, sometimes I will just like, you know, in when I’m in the bath, I definitely do it probably at least once a week now. – Charlotte, Average Risk

Women at increased risk expressed a keenness to adopt early screening. Some of the women interviewed had already received early screening, while others were awaiting this but had made the decision to take this up when offered. This was the most accepted option for monitoring risk following their risk assessment.

Really happy, [. . .]I think it sort of reassures me that there’s going to be an additional thing in place as well as me sort of being aware of my own body in the meantime. So, yeah, I feel really positive that I wouldn’t have had access to any of this information or be eligible for that without having done this research. – Leah, Increased Risk

When women were presented with the idea that their risk estimate could change at the end of the study, there were some concerns that should their risk reduce, they would no longer be able to access this screening. This concern raised anxiety for some women, with them expressing discomfort or opting to take up private screening if they were no longer eligible.

In comparison with earlier screening, women were more reluctant to commence risk-reducing medication, expressing concern about the potential quality of life changes that may occur with taking hormonal medications. However, some women were open to trialling the medication with the decision to continue informed by experience of side effects.

I don’t know if I want to take daily medication. But I think I’ll probably try it and see if it doesn’t affect me too badly, because some of the side-effects are quite – sounded pretty bad in some people. – Natalie, Increased Risk

### Improving the offer of breast cancer risk assessment

This theme describes the recommendations women had for improving the offer of breast cancer risk assessment in the future.

#### Streamlining delivery of breast cancer risk assessment

All participants considered invitation to take part in breast cancer risk assessment via their general practice to be suitable. For many, this encouraged participation as general practitioners (GPs) were perceived as a familiar and trusted source of health information.

I will accept it more from my GP than receiving it from like a hospital or a university because my GP, I trust my GP, I know my GP, I visited my GP many times. – Nadia, Increased Risk

However, many women at increased risk were unhappy with the process of being referred to the family history clinic via their GP as it was slow, and some women had to contact their GP on multiple occasions to chase the referral. Participants were concerned that this part of the care pathway could result in women being lost to follow-up. They preferred to be automatically referred upon receiving their risk estimate, with an appointment sent to them directly.

I think if you’re at an increased risk, so that triggering an appointment rather than me then having to go to my GP and ask to be referred to then have the appointment . . . I think if that had the date of your appointment, if that was sort of automatic and you had that appointment date at the same time then it would be helpful. – Leah, Increased Risk

The other issues about delivery were practical barriers to attending the risk assessment appointment at a hospital in South Manchester. Several participants travelled a substantial distance, with some experiencing parking issues. Furthermore, participants suggested offering more flexible appointment times, including evenings and weekends to allow those in full-time work the opportunity to attend without using leave.

I know a lot of people are so busy that they might think, hmm, I work full-time, I do – you know, like how would I get there, kind of thing whereas I felt like I was – you know, I was lucky enough that I was – I had free time and I’m near the hospital. – Maeve, Increased Risk

Furthermore, one participant raised concerns regarding the utilisation of facilities designated for cancer patient care.

I’m well aware they’re very busy and I’d rather them prioritise actual cancer patients than myself. – Lauren, Increased Risk

#### Addressing knowledge deficits

Although participants reported having all the information required to make a decision about participating in breast cancer risk assessment, it became apparent that a few women had misconstrued the purpose of the mammogram despite this being explicitly communicated in the participant information sheet. These women anticipated that the study would provide definitive diagnostic results regarding the presence of breast cancer, mistakenly equating the risk assessment procedure with routine cancer screening. Some women would have preferred the mammogram to be capable of both density and diagnostic assessment and were unclear why this could not be the case. Women reported that any confusion about the mammogram’s purpose was clarified by the research nurses with all participants understanding its purpose prior to undergoing the procedure.

And foolishly I thought it might help me to discover if I’ve got breast cancer already, but I didn’t know that, that has been explained to me at the actual appointment by the lovely nurse who seen me, that the radiation isn’t as strong, so – it was slightly different than I thought. – Megan, Average Risk

Some women did not identify with the risk category they received while others reported that their risk estimate did not match their expectations. Participants often cited the absence of family history of breast cancer as a reason for being surprised at receiving an increased risk estimate.

I feel like I’m a bit lower than average because I’m sort of like a healthy weight and exercise and don’t drink and smoke and stuff like that. So it said that I’m average, but then I’m like, I’m probably a little bit lower than average. – Jennifer, Average Risk

The written risk feedback was considered too complex, with the statistical and genetic terminology particularly difficult to comprehend. Additionally, the format in which the risk estimates were conveyed, particularly through tables and graphs, was deemed unhelpful by some of the participants. Some women desired more information in the risk feedback letter about how their risk result was calculated. April expressed some concern about the accuracy of her risk result due to her ethnicity, as non-white European women have been understudied historically.

I think in that letter, I don’t think they actually mentioned that the potential reasons why, so yeah I don’t think it was clear enough. . . I guess if you don’t have a data, you don’t have data of like non white Caucasian women, then that probably is the explanation, but yeah. But I was happy to wait for the appointment with the breast cancer – the family history clinic, to find out a bit more. – April, Increased Risk

One participant described an interaction with a new member of the research team whereby she was disclosing her family history of cancer (her mother has a diagnosis of breast cancer), and she felt the way that ‘strong family history’ was communicated to her diminished her situation.

Even if they don’t have, like, loads of aunties and sisters with it or whatever, having one relative is enough, and I think that that is probably, again, for a lot of women, like, a motivating factor. And so, to just then be told, like, ‘Oh well, that’s not a strong family link,’ it’s like, well, it is a strong family link, it’s just maybe not in the way that you think it is. – Lauren, Increased Risk

This demonstrates how a layperson’s definition of a strong family history and that of the research team may differ.

## Discussion

This is the first study to explore the experiences of receiving breast cancer risk estimates outside an organised breast screening programme by women aged 30–39 years without a strong family history of breast cancer. Overall, women found it acceptable to undergo breast cancer risk assessment, with very few reports of anxiety during or after participation. All women valued the opportunity to learn more about risk reduction and for increased risk women, access to screening and preventive measures. The professionalism and supportive nature of staff was seen as important in ensuring a positive and informed experience. The processes of invitation, risk assessment and feedback processes were largely acceptable. However, knowledge deficits, particularly in relation to risk comprehension, were noted. Participants suggested ways to improve the offer of breast cancer risk assessment including simplified risk feedback, flexible appointments and a more efficient referral process to secondary care for women at increased risk.

## Limitations

The use of two interviewers is considered a strength of this study. Using multiple interviewers reduces individual bias, as each interviewer brings unique perspectives and interpretations to the data collection process, contributing to a richer and more diverse dataset.^41^ It also enhances credibility and trustworthiness of the data, as consistency across different interviewers’ findings can validate the robustness of the themes identified during analysis.^42^ Interviews were also analysed by multiple members of the team, enabling a more balanced interpretation. This collaborative approach also promoted reflexivity, allowing researchers to critically reflect on their assumptions and improve the quality of their analysis during discussions.

However, of the 22 women interviewed, only 5 were from deprived areas and only 6 participants did not identify as white British. This may make it difficult to apply the findings to a broader, more diverse population, who might have different barriers or facilitators to engaging in risk assessment. Although purposive sampling was utilised in this study, it was challenging to ensure a fully diverse sample of women as many who were invited did not choose to participate in an interview. Furthermore, women who took part in BCAN-RAY, by virtue of joining and agreeing to have their risk assessed, could be more likely to be receptive to breast cancer risk assessment than those who were offered and did not join BCAN-RAY, leading to overestimations of acceptability. Therefore, the experience of those who were ambivalent or opposed to risk assessment has not been captured.

Moreover, a notable time interval occurred between participants completing the risk assessment process and attending interviews. This delay was attributed to factors such as awaiting risk feedback and, for participants identified as at increased risk, awaiting referral to the family history clinic. While this could be considered a limitation of the study, it ensured that participants’ recollections were focused on the elements of the process that were most salient to them.

Additionally, it should be noted that this study was conducted within a highly specialised centre with substantial expertise and resources dedicated to breast cancer risk assessment. Such conditions may not be replicated in other settings, where differences in infrastructure, staffing levels and training could influence both the feasibility and outcomes of implementing similar approaches. As a result, the generalisability of these findings may be limited, since patient experiences and effectiveness of the risk assessment process could vary considerably across institutions with differing capacities.

## Relevance to existing literature

In line with previous research in older women,^25,43,44^ breast cancer risk assessment was found to be acceptable with no major adverse effects from receiving risk estimates reported. The lack of anxiety reported may be due to the availability of research staff support throughout the process. Approximately 1 h was set aside for the risk assessment and risk review appointments allowing discussion of any concerns or questions. It is important to acknowledge that the present study was conducted at a centre of excellence for breast cancer risk prediction, and prevention research and other centres may not have the same level of resource or expertise in breast cancer risk. This is a potential challenge to scaling up this risk assessment process as having access to dedicated and skilled staff members was a key facilitator to the success of this project. Differing levels of staff availability and training may impact the patient experience and as such could lead to different outcomes than those highlighted in this study.

Staff support was also important to address knowledge deficits, especially to facilitate understanding of risk. In the present study, some women did not identify with the risk result received, particularly when receiving an increased risk estimate in the absence of a strong family history of breast cancer. This is consistent with previous findings that women’s ability to internalise their clinically derived risk is influenced by personal risk appraisals and expectations.^45^ The presence of family history has been found to dominate screening age women’s breast cancer risk perceptions.^46^ Therefore, women with some family history may not expect an average risk estimate.

Some women expressed heightened breast awareness following participation in the BCAN-RAY study. In a previous qualitative study, women aged 30–39 years reported uncertainty about what they should do and lacked confidence in performing checks due to limited knowledge about what to look and feel for.^27^ There is a need to define the best strategy, in terms of recommended behaviours, for breast awareness, so women know what to do. Only the minority (about 20%) will receive an increased risk result, so for the majority, the only recommendation will be breast awareness. Previous literature suggests informational support will be required.

## Implications and future research directions

While participants found the process acceptable, key barriers to comprehension and accessibility must be addressed to optimise future implementation.

First, staff support was identified as important in clarifying study procedures and facilitating risk comprehension, with written materials alone proving insufficient for many women. To improve risk communication, future research should prioritise developing informational materials in collaboration with women, using a co-design process to ensure the content is tailored to their specific concerns and preferences.^32^ An improvement in the accessibility of the risk results and materials provided in BCAN-RAY may help mediate any difficulties with scaling up this project, as women may be less reliant on staff support to understand the important details of this process. Improved materials would also support women’s understanding of the purpose of the mammography and the guidance surrounding breast awareness, further supporting the few women who misconstrued these areas.

Second, issues with risk comprehension were evident, with some participants not identifying with their risk estimates or struggling with technical terminology. Women’s understanding of risk was not explored in depth within this study as the primary focus was to assess acceptability of risk assessment processes; nevertheless, some participants reported comprehension difficulties. Given these findings, future research should further explore how women understand, appraise and interpret their breast cancer risk results. Investigating cognitive and emotional responses to different risk communication strategies will be essential to refining educational resources and improving accessibility. Using three or four risk categories, as seen in studies like PROCAS,^47^ may enhance comprehension compared with a two-category system, allowing for more nuanced risk communication. Investigating cognitive and emotional responses to different risk communication strategies will be essential to refining educational resources and improving accessibility. In addition to this, materials provided may benefit from being adapted to communicate risk in easy access formats or via telehealth options such as iPrevent.^48^

Third, logistical barriers to mammogram attendance were identified. Many participants found accessing the risk assessment site challenging due to parking issues and limited appointment flexibility, alongside sharing concerns about using facilities needed for cancer patients. The acceptability of alternative delivery models, such as integrating risk assessment within primary care or implementing mobile screening units – similar to lung health checks – should be investigated as they may enhance accessibility and uptake. In previous research, primary care providers have reported reservations about being involved in the management of women identified as increased risk.^47^ Furthermore, women who participated in PROCAS have reported not being in favour of receiving risk feedback and screening and prevention advice from GPs in the future due to difficulties obtaining risk-reducing medication.^49^ Similarly, previous research highlighted that women valued personalised prevention information, but they expressed concerns about GP expertise and time to deliver complex risk assessments, highlighting the need for specialist involvement.^50^ Therefore, it would be worthwhile to investigate automatic referrals to the family history clinic for women who are increased risk to avoid primary care becoming a bottleneck for referrals.

Generalising breast cancer risk assessment across different health care settings requires adapting risk prediction models, training professionals and integrating digital solutions. Studies highlight the importance of validating risk prediction models in diverse populations to ensure accuracy.^51^ Simplified models that rely on widely available clinical and demographic data can help centres with fewer resources implement effective risk assessment.^51^ In addition to this, it is important to investigate where telehealth options may support with risk assessment. Specifically, web-based tools such as iPrevent may support in addressing potential gaps in care. These support tools can empower women to make decisions about their care based on their risk result and may be easier to access for some than a family history clinic.^52^

As young black women are at higher risk of aggressive subtypes of cancer developing pre-screening age, and women of south Asian descent are at higher odds of being diagnosed with late-stage breast cancers,^53^ further research exploring the acceptability of breast cancer risk assessment in an ethnically and socio-economically diverse sample is needed. This is vital to ensure a more cognisant approach to implementation, taking into account the needs of different groups.

## Conclusion

Breast cancer risk assessment amongst younger women was generally acceptable, with no evidence of significant anxiety during and after participation. Participants valued breast cancer risk assessment as an opportunity to be proactive about their own health. Despite identified knowledge gaps and logistical barriers, these challenges present opportunities for refinement, including clearer risk communication and automated referrals for women at increased risk, to increase the likelihood of successful implementation in the future.

## Supplemental Material

sj-docx-1-whe-10.1177_17455057261435107 – Supplemental material for Acceptability of breast cancer risk assessment amongst general population women aged 30–39 years: A qualitative study Supplemental material, sj-docx-1-whe-10.1177_17455057261435107 for Acceptability of breast cancer risk assessment amongst general population women aged 30–39 years: A qualitative study by Rosie Horton, Sarah Hindmarch, Sacha J. Howell, Juliet A. Usher-Smith, Louise Gorman, D. Gareth Evans and David P. French in Women's Health

sj-docx-2-whe-10.1177_17455057261435107 – Supplemental material for Acceptability of breast cancer risk assessment amongst general population women aged 30–39 years: A qualitative study Supplemental material, sj-docx-2-whe-10.1177_17455057261435107 for Acceptability of breast cancer risk assessment amongst general population women aged 30–39 years: A qualitative study by Rosie Horton, Sarah Hindmarch, Sacha J. Howell, Juliet A. Usher-Smith, Louise Gorman, D. Gareth Evans and David P. French in Women's Health

sj-docx-3-whe-10.1177_17455057261435107 – Supplemental material for Acceptability of breast cancer risk assessment amongst general population women aged 30–39 years: A qualitative study Supplemental material, sj-docx-3-whe-10.1177_17455057261435107 for Acceptability of breast cancer risk assessment amongst general population women aged 30–39 years: A qualitative study by Rosie Horton, Sarah Hindmarch, Sacha J. Howell, Juliet A. Usher-Smith, Louise Gorman, D. Gareth Evans and David P. French in Women's Health

sj-docx-4-whe-10.1177_17455057261435107 – Supplemental material for Acceptability of breast cancer risk assessment amongst general population women aged 30–39 years: A qualitative study Supplemental material, sj-docx-4-whe-10.1177_17455057261435107 for Acceptability of breast cancer risk assessment amongst general population women aged 30–39 years: A qualitative study by Rosie Horton, Sarah Hindmarch, Sacha J. Howell, Juliet A. Usher-Smith, Louise Gorman, D. Gareth Evans and David P. French in Women's Health
