# Supplementary material for: Acceptability of breast cancer risk assessment amongst general population women aged 30–39 years: A qualitative study
Source: Womens Health (Lond). 2026 Apr 24;22:17455057261435107. doi: 10.1177/17455057261435107 (PMC13125794; doi:10.1177/17455057261435107)
Supplement: sj-docx-2-whe-10.1177_17455057261435107 – Supplemental material for Acceptability of breast cancer risk assessment amongst general population women aged 30–39 years: A qualitative study [file sj-docx-2-whe-10.1177_17455057261435107.docx]

**Interview schedule**

***For ethics committee and researchers:*** *This interview aims to understand women’s experience of and views about having their breast cancer risk assessed in the BCAN-RAY study. It also seeks to explore whether the materials and pathways that have been developed for BCAN-RAY are suitable for women. Furthermore, women will be asked to comment on how the offer of breast cancer risk assessment could be improved in terms of delivery and provision of support and information.*

**Introduction**

Thank you for agreeing to take part in this interview. The interview may last up to 60 minutes, but we can stop at any time if you need to. Just to remind you, you do not need to answer all the questions and there are no right or wrong answers. We just want to hear your views.

Is there anything you want to ask before we start? *[opportunity for questions]*

Please could you confirm that you are happy to take part in the study and you agree to the interview being audio recorded *[await confirmation]*

*[switch audio recorder on]*

**OPENING QUESTIONS**

As you know, we are interested in what women think about the offer of finding out their breast cancer risk as part of the BCAN-RAY study. To start, can you tell me anything about whether breast cancer risk is something you have thought about before being invited to join the BCAN-RAY study?

I understand you were invited to have your breast cancer risk assessed; can we go back to that point and tell me what that was like? What did you think at that point? How did you feel?

How did you make the decision to take part in breast cancer risk assessment?
Prompts:

- How did you receive the invite (as a letter from GP practice if no recall)? What do you think about receiving it that way (different format preferred)? How do you think that influenced your decision to have your breast cancer risk assessed?
- Can you tell me anything about why you wanted to know your risk? Anything personal to you?
- Were there are aspects of the BCAN-RAY study that made you question whether to take part (any concerns)?
- (if not already come up) When you were deciding, did you discuss it with anyone (friend / family / study team / GP)?
- Did you feel you had all the information you needed to make a decision about whether to take part? If not, what would have been helpful to know?
- Did you understand what was involved when you made the decision to participate?

**QUESTIONS RELATING TO RISK ASSESSMENT PROCESS**

Can you tell me what you had to do once you joined the study? Could you tell me about what happened when you had your breast cancer risk assessed?

Probes: What was it like / can you tell me anything about it

Prompts:

- Completing the risk factors questionnaire e.g. how easy was it to access, can you remember what it was asking you to do, were any questions unclear, ability to answer the questions more generally, did you find any questions uncomfortable to answer, did you get any support to help with this part of the study
- What happened once you completed the questionnaire? What was that time-period like?
- Attending the appointment at the hospital (spit sample, mammogram) e.g. what did you think about how the appointment was arranged, what was the mammogram procedure like, is there anything that could have been improved
- Waiting for the risk feedback results (up to 16 week turnaround) e.g. how were you feeling during this time, do you recall having any particular thoughts or feelings during this time, what did you think about the length of time you had to wait, did you look for any information related to breast cancer during this time
- Receiving the risk feedback (a letter or email if no recall) e.g. what did you think about receiving the feedback in this way
- Contents/wording of the letter (thoughts, feelings and understanding) e.g. did the feedback you received match your expectations in terms of what you thought you would be told, could the risk information you received be improved in any way (content, format)
- Personal meaning of risk category received e.g. what do you remember about your risk result, how would you describe the risk, how did receiving your risk result make you feel, was it something you expected, how do you feel about your risk today/now
- Discussing risk feedback with others (friends / family / healthcare professionals)
- Did you talk about your risk feedback with anyone in the study team / outside the study team? If yes/no, why? What did you discuss?
- What did you think of the support provided at this point?
- (increased risk) Experience of risk consultation
- What did you think about the option to receive an appointment to discuss your risk if it was increased?
- Can you tell me about your experience of the risk consultation? Could it have been improved in any way?
- How do you feel about the factors that contributed to your risk?

After you received your risk feedback, did you do anything differently that you thought might reduce your breast cancer risk? How informed do you feel now about how breast cancer risk can be reduced?

Prompts:

- (all) Health behaviours
- (increased risk) Recommendation to contact medical doctors to discuss risk reducing medication / additional screening
- (increased risk) Deciding whether to have additional screening
- (increased risk) Deciding whether to have risk reducing medication

Looking back, was there anything that caused any concerns during the risk assessment process? Is there anything you would have preferred to happen in a different way?

Looking back, how do you feel about having made the decision to take part in breast cancer risk assessment?

Prompts:

Was there any information that you felt like it would have been beneficial to have had at any point, that you didn’t get?

The way breast cancer risk is calculated changes over time as we learn more about new risk factors. As we are trying to find out whether using a low dose mammogram helps to identify younger women at risk of developing breast cancer, towards the end of the study you will receive updated risk feedback. At this point, your risk might change. What are your thoughts about this? Why?

We are trying to figure out whether introducing a breast cancer risk assessment service for women aged 30 to 39 years is a good or bad idea. What are your thoughts about this? Why? Would you recommend a breast cancer risk assessment service to friends and family members of a similar age?

**Finishing comments**

Thanks for your time today. We do really appreciate it.

- Is there anything else you want to add?
- Is there anything you thought you would talk about today which you haven’t had a chance to say and want to mention?
- What’s the most important thing about your breast cancer risk assessment experience that you have told me today?
- Do you have any questions for me?

Thanks again. The interview will be typed up by a partner transcription company we use. When this is done, we will remove anything you have said that could identify you such as names or places and you will be given a fake name. If you have any questions feel free to contact the research team at any time *[point out contact details]*. Ask for shopping voucher preference (Amazon, Love2Shop or Blackwells) and email address for e-voucher to be sent to.
