# Supplementary material for: Acceptability of breast cancer risk assessment amongst general population women aged 30–39 years: A qualitative study
Source: Womens Health (Lond). 2026 Apr 24;22:17455057261435107. doi: 10.1177/17455057261435107 (PMC13125794; doi:10.1177/17455057261435107)
Supplement: sj-docx-3-whe-10.1177_17455057261435107 – Supplemental material for Acceptability of breast cancer risk assessment amongst general population women aged 30–39 years: A qualitative study [file sj-docx-3-whe-10.1177_17455057261435107.docx]

**PPIE group feedback:**

The findings across the four themes were presented to 2 representatives of the PPIE group to highlight key considerations for improving risk assessment processes. During this session, the results were shown to the group and then they were asked what the implications of this are and how we can best apply these findings to improve future trials. The group first highlighted the importance of training healthcare professionals in soft skills to enhance patient experience, as preparation and empathy can shape outcomes and reduce feelings of dismissal, particularly for individuals concerned about factors such as weight. The group also reaffirmed the importance of clear communication to address misconceptions about risk assessments being diagnostic, mitigate health anxiety, and improve accessibility of invites and results. It was suggested that it would be beneficial to utilise lay summaries supplemented by detailed options for those seeking more information. The group shared concerns about feedback relating to behaviour changes, suggesting that discussing factors such as weight could potentially lead participants to become defensive. They instead suggested a focus on lesser-known risks to avoid defensiveness, while transparent information on risk calculations and medication options—including costs and trial opportunities—would better support informed decision-making, especially during financial strain. Finally, practical adjustments such as neutral settings, mobile units, flexible appointment times, and streamlined GP referral processes would increase accessibility and reduce barriers, particularly for vulnerable groups. These findings reiterate the need for patient-centred approaches that prioritise clarity, inclusivity, and support mechanisms to enhance engagement and outcomes.
